# Application of machine learning algorithms to identify people with low bone density

**DOI:** 10.3389/fpubh.2024.1347219

**Published:** 2024-04-25

**Authors:** Rongxuan Xu, Yongxing Chen, Zhihan Yao, Wei Wu, Jiaxue Cui, Ruiqi Wang, Yizhuo Diao, Chenxin Jin, Zhijun Hong, Xiaofeng Li

**Affiliations:** ^1^Department of Epidemiology and Health Statistics, Dalian Medical University, Dalian, China; ^2^The Health Management Center, The First Affiliated Hospital of Dalian Medical University, Dalian, Liaoning, China

**Keywords:** low bone density, osteoporosis, machine learning, blood biochemical indicators, National Health and Nutrition Examination Survey

## Abstract

**Background:**

Osteoporosis is becoming more common worldwide, imposing a substantial burden on individuals and society. The onset of osteoporosis is subtle, early detection is challenging, and population-wide screening is infeasible. Thus, there is a need to develop a method to identify those at high risk for osteoporosis.

**Objective:**

This study aimed to develop a machine learning algorithm to effectively identify people with low bone density, using readily available demographic and blood biochemical data.

**Methods:**

Using NHANES 2017–2020 data, participants over 50 years old with complete femoral neck BMD data were selected. This cohort was randomly divided into training (70%) and test (30%) sets. Lasso regression selected variables for inclusion in six machine learning models built on the training data: logistic regression (LR), support vector machine (SVM), gradient boosting machine (GBM), naive Bayes (NB), artificial neural network (ANN) and random forest (RF). NHANES data from the 2013–2014 cycle was used as an external validation set input into the models to verify their generalizability. Model discrimination was assessed via AUC, accuracy, sensitivity, specificity, precision and F1 score. Calibration curves evaluated goodness-of-fit. Decision curves determined clinical utility. The SHAP framework analyzed variable importance.

**Results:**

A total of 3,545 participants were included in the internal validation set of this study, of whom 1870 had normal bone density and 1,675 had low bone density Lasso regression selected 19 variables. In the test set, AUC was 0.785 (LR), 0.780 (SVM), 0.775 (GBM), 0.729 (NB), 0.771 (ANN), and 0.768 (RF). The LR model has the best discrimination and a better calibration curve fit, the best clinical net benefit for the decision curve, and it also reflects good predictive power in the external validation dataset The top variables in the LR model were: age, BMI, gender, creatine phosphokinase, total cholesterol and alkaline phosphatase.

**Conclusion:**

The machine learning model demonstrated effective classification of low BMD using blood biomarkers. This could aid clinical decision making for osteoporosis prevention and management.

## 1 Introduction

Osteoporosis, the most prevalent metabolic bone disorder, is characterized by low bone mass, microarchitectural deterioration, fragility, and increased fracture risk (1–3). The growing older adult/adults population has contributed to rising osteoporosis prevalence globally - currently estimated at 19.7% (4–6). Fractures in six EU nations may increase from 2.7 million in 2017 to 3.3 million by 2030, with costs rising by 27% to $37.5 billion (7). Thus osteoporosis imposes substantial socioeconomic burdens worldwide. However, its subtle onset often delays diagnosis until fractures occur (8). Effective screening and early interventions are critical for prevention. In other words, it is important to screen for osteopenia and osteoporosis in the general population, in order to enable timely interventions to prevent fragility fractures. Dual-energy X-ray absorptiometry remains the gold standard for measuring BMD (9). However, the need for skilled technicians and radiation exposure limit its widespread use (10, 11). Since some blood biomarkers have shown modest correlations with osteoporosis and are easily obtained, this study aimed to develop biomarker-based models to identify those with low BMD (12–14). Machine learning, an important artificial intelligence tool, discovers patterns in big datasets via complex algorithms (15). Advancements in healthcare big data have expanded ML applications (16). The purpose of this study is to utilize the data from the National Health and Nutrition Examination Survey (NHANES) database to build models and test them using six machine learning algorithms, namely, logistic regression (LR), support vector machine (SVM), gradient boosting machine (GBM), naive Bayesian (NB), artificial neural network (ANN), and random forest (RF), which were modeled and tested to compare the accuracy of several methods in predicting low bone density in the test set, and to explore the application value of machine learning algorithms in low bone density prediction and auxiliary diagnosis.

## 2 Materials and methods

### 2.1 Dataset source

The National Health and Nutrition Examination Survey (NHANES) database was selected for this study. The NHANES is a program designed by the National Center for Health Statistics (NCHS) to assess the health and nutritional status of the U.S. population by surveying a national sample of 5,000 citizens annually since 1999. NHANES protocols were approved by the NCHS Research Ethics Review Board with written informed consent obtained from all participants (17).

### 2.2 Participants

In this study, NHANES data for the cycle 2017–2020 was selected as the internal validation set, and NHANES data for the cycle 2013–2014 was used as the external validation set, excluding participants younger than 50 years of age and participants with missing or invalid Femoral neck BMD data in Dual-Energy X-ray Absorptiometry – Femur.

### 2.3 Variable selection and definition

Based on previous literature (18, 19) and the purpose of the study, the following four components of variables were included: (a) Demographic information: age, gender, race and education, marital status, poverty index. (b) Examination data: Dual-Energy X-ray Absorptiometry - Femur (Femoral neck BMD), body mass index (BMI). (c) Laboratory data: Standard Biochemical Profile, Plasma Fasting Glucose, HDL, LDL & Triglycerides, Total Cholesterol, Complete Blood Count, Glycohemoglobin. (d) Questionnaire information: Osteoporosis, Alcohol Use, Blood Pressure &Cholesterol, Diabetes, Smoking-Cigarette Use. Alcohol use was defined as having ever had 4/5 drinks or more per day; smoking was defined as having smoked at least 100 cigarettes in one’s lifetime; having ever been told that one has high blood pressure or is on prescription medication for high blood pressure was defined as high blood pressure; having ever been told that one has diabetes or is on insulin or glucose-lowering medication was defined as diabetes; and history of personal osteoporosis or fracture is defined as having at least one of the following: ever had a hip, wrist, spine or other fracture; been told by a doctor that you have osteoporosis. Parental history of osteoporosis or fracture was defined as having at least one of the following: self-reported fracture of a parent; parent had been told that he or she had osteoporosis.

### 2.4 Evaluation of low bone density

Bone mineral density (BMD) measurements in the NHANES database were primarily determined using dual-energy X-ray absorptiometry (DXA). In 2017–18, the femur scans were acquired on Hologic Discovery model A densitometers (Hologic, Inc., Bedford, Massachusetts), using software version Apex 3.2. Bedford, Massachusetts, using software version Apex 3.2. In 2019-March 2020, the femur scans were acquired on Hologic Horizon model A densitometers (Hologic, Inc., Bedford, Massachusetts), using software version Apex version 5.6.0.5. The 2013–2014 femur scans were acquired on Hologic QDR-4500A fan-beam densitometers (Hologic, Inc., Bedford, Massachusetts) using software version Apex 3.2. All scans were analyzed with Hologic APEX version 4.0 software. In this study, the BMD of the femoral neck was chosen as a criterion because it has been proposed as a reference skeletal site for defining osteoporosis in several epidemiologic studies (11). The diagnosis of primary osteoporosis and osteopenia is mainly based on the T-value obtained after the calculation of BMD measurements (20). *T*-value = bone mineral density of the study population – mean value of bone mineral density of the reference group (age group of peak bone mineral density)/standard deviation of that reference age group (World Health Organization recommendations use bone mineral density data of non-Hispanic white women aged 20–29 years from NHANES III as the reference group).

*T*-value ≥ −1: healthy −2.5 < *T*-value < −1: osteopenia *T*-value ≤ −2.5: osteoporosis

Both conditions, osteopenia and osteoporosis, are considered to be low bone mineral density (21), and are therefore defined as low bone mineral density when either of the following is met: (1) femoral neck *T*-score < −1 (2) patient said “yes” to the question: Has a doctor ever told you that you had osteoporosis, sometimes called thin or brittle bones?

### 2.5 Statistical analysis

#### 2.5.1 Data cleaning

Participants aged ≥50 years with complete femoral neck BMD data were included. Due to substantial missingness and outliers, data preprocessing was performed. We assigned “NA” to the data with “7, 9, 77, 99,” deleted the variables with more than 30% missing values (22, 23), and used the MI package in the R software to perform multiple interpolation for the variables with less than 30% missing values. Summary statistics were calculated following imputation. Normally or near-normally distributed continuous variables were presented as mean ± standard deviation and compared between groups by independent t-tests. Non-normally distributed continuous data were expressed as median (interquartile range) and compared using non-parametric tests. Categorical variables were presented as *n* (%) and compared via chi-squared tests.

#### 2.5.2 Feature selection

In this study, Lasso (Least Absolute Shrinkage and Selection Operator) feature selection was performed using the ‘glmnet’ package in the R software. By adding an L1 regularization term to the least squares function, LASSO forces some coefficients to zero, effectively removing those variables from the model. An important tuning parameter in LASSO is λ (λ ≥ 0), controlling the degree of coefficient shrinkage. When λ = 0, LASSO is equivalent to ordinary linear regression. This study performs 10-fold cross-validation through the ‘cv.glmnet’ function, that is, the data are randomly divided into 10 groups, nine of which are used as the training set and one as the test set, and one extreme value of λ is generally selected for the training set, and then the parameters obtained from the training set are used for the prediction of the remaining set of data, and this process is repeated for 10 times, and the optimal value of λ is finally determined by the mean-square error obtained from the calculation of the results of the 10 predictions. Under this function, there are usually two choices for the optimal λ value, one is λ.min, the value of λ that minimizes the cross-validation error; the other is λ.1se, which keeps the cross-validation error within one standard error. The choice of the optimal λ varies from study to study depending on the specifics of the study and the purpose of the study. In addition, Lasso performs well in coping with the problem of the existence of multiple covariates among variables, and the independent variables in this study are mainly common blood biochemical indexes in clinics, and there is often the effect of multiple covariates among these variables, while Lasso regression can effectively deal with the problem of covariates by forcing some of the coefficients to be contracted to zero, which improves the stability and interpretability of the models (24).

#### 2.5.3 Modeling and evaluation

In machine learning, there are four main methods: supervised learning, unsupervised learning, semi-supervised learning and reinforcement learning. The goal of this study is to categorize the population with normal bone density and the population with low bone density. Since this is a classification problem, the use of supervised learning algorithms is most appropriate (25). Therefore, six commonly used supervised learning algorithms, logistic regression (LR), support vector machine (SVM), gradient boosting machine (GBM), naive Bayes (NB), artificial neural network (ANN), and random forest (RF), were used to construct the model in this study. The internal validation dataset was randomly divided into training set and test set according to the ratio of 7:3. During the model training process, 10-fold cross-validation was used to select and adjust the model parameters. Then, 30% of the test dataset was input into the trained model for prediction. Additionally, NHANES data from 2013 to 2014 was entered into the model for external validation. The model performance was evaluated in terms of model differentiation ability, calibration ability and clinical application value. The area under the receiver operating characteristic curve (ROC) (AUC), accuracy, sensitivity, specificity, precision and F1 score were utilized to assess the discriminative ability of the model. Calibration ability of the model was assessed using calibration curves. The clinical applicability of the models was assessed by decision curve (DCA), and the confusion matrices of several models were visualized to provide a more intuitive understanding of the classification ability of the models.

#### 2.5.4 Evaluation of the importance of variables

SHAP (SHapley Additive exPlanation) is a post-hoc explanation framework for machine learning models based on game theory (26). It quantifies the importance of each feature in the model by calculating the contribution value, known as the Shapley value, for each feature towards the predicted outcome. This study utilizes the SHAP method to enhance the interpretability and transparency of the model. The data analysis process was conducted using R 4.3.1 and Python 3.11.3, and a significance level of *p* < 0.05 was considered statistically significant.

## 3 Results

### 3.1 Baseline characteristics

**Figure 1:**
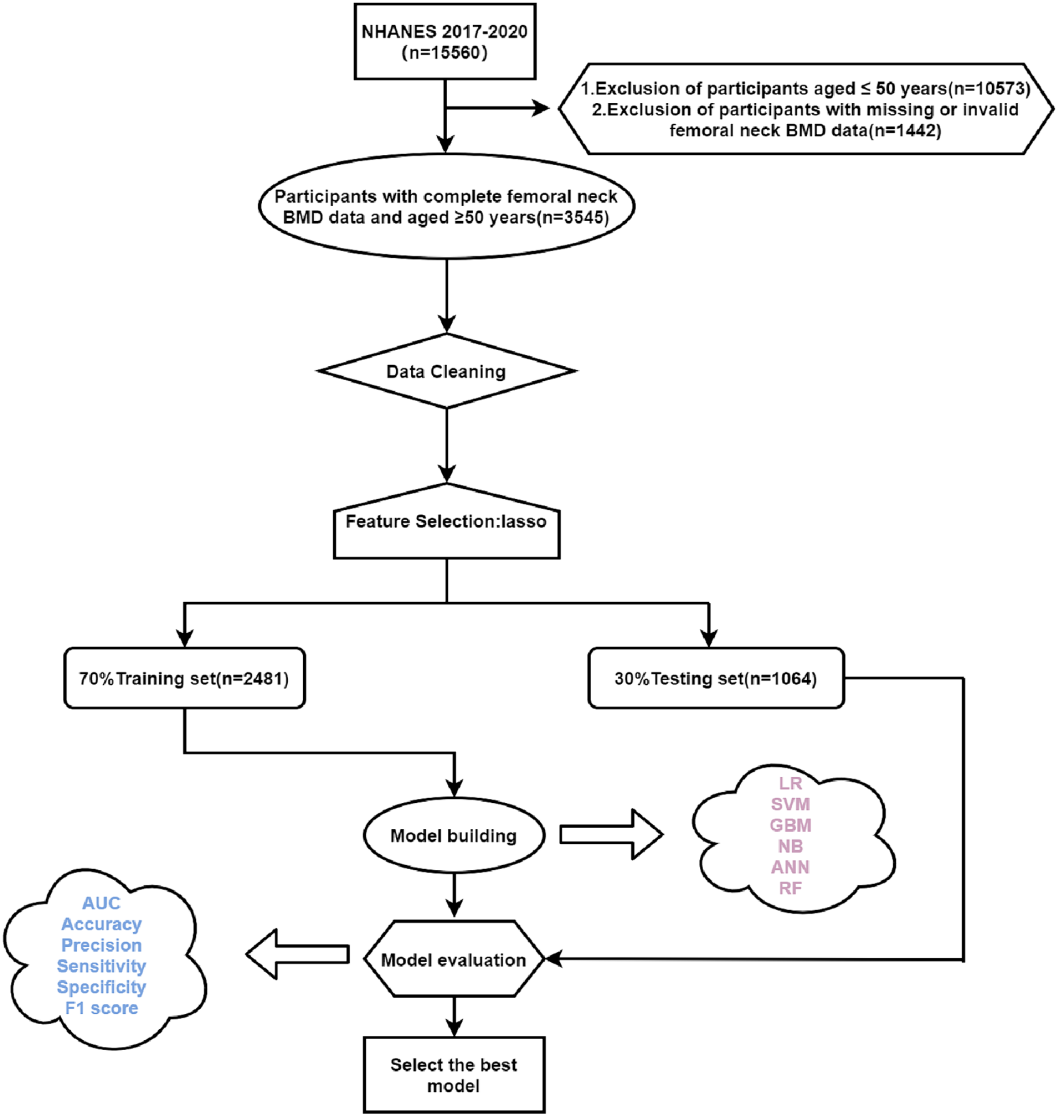
Flow chart of this study. LR, logistic regression; SVM, support vector machine; GBM, gradient boosting machine; NB, naive Bayesian; ANN, artificial neural network; RF, random forest; Lasso, Least Absolute Shrinkage and Selection Operator.

Based on the inclusion and exclusion criteria, a total of 3,545 study participants who were ≥50 years of age and had complete femoral neck BMD data were included in the internal validation set of this study (Figure 1). The baseline information of the study subjects is shown in Table 1, of which 1870 were in the normal BMD group and 1,675 in the low BMD group, and a total of 60 initial variables were included after deletion of variables with more than 30% of missing values (Fasting Glucose, LDL-Cholesterol, and Triglyceride); among the demographic factors, lifestyle factors and past medical history, it can be seen that compared to the normal BMD group, the low BMD group was more likely to be older, female, non-Hispanic white or other race, widowed/divorced/separated, no history of smoking and alcohol consumption, lower BMI, no diabetes, and have a personal and parental history of osteoporosis and fracture; among the blood biochemical indexes, the mean values of direct HDL-Cholesterol, Total Cholesterol, Segmented neutrophils percent, Mean cell volume, Mean cell hemoglobin, Alkaline Phosphatase (ALP) were greater in the low bone density group than in the normal bone density group, while the mean values of Red blood cell count, Hemoglobin, Hematocrit, Glycohemoglobin, Alanine Aminotransferase (ALT), Creatine Phosphokinase (CPK), Creatinine, Globulin, Glucose, Gamma Glutamyl Transferase (GGT), Total Protein, Uric acid were smaller than those of the normal BMD group (*p* < 0.001). The external validation set screened 3,127 study participants, of whom 1,796 were in the normal BMD group and 1,331 in the reduced BMD group, and the baseline information table is shown in Supplementary Table S1.

**Table 1:** Comparison of general characteristics of the group with normal bone mineral density and the group with low bone mineral density.

	**Normal bone density** ***N* = 1870**	**Low bone density** ***N* = 1,675**	***P***
Age(year)	62.5 (8.49)	66.6 (9.09)	<0.001
Gender (*n*, %)			<0.001
Male	1,234 (66.0%)	642 (38.3%)	
Female	636 (34.0%)	1,033 (61.7%)	
Race (*n*, %)			<0.001
Mexican American	192 (10.3%)	141 (8.42%)	
Other Hispanic	214 (11.4%)	173 (10.3%)	
Non-Hispanic White	599 (32.0%)	773 (46.1%)	
Non-Hispanic Black	630 (33.7%)	280 (16.7%)	
Other Race	235 (12.6%)	308 (18.4%)	
Education level (*n*, %)			0.641
Less than 9th grade	172 (9.20%)	156 (9.31%)	
9–11th grade	190 (10.2%)	173 (10.3%)	
High school graduate/GED or equivalent	473 (25.3%)	426 (25.4%)	
Some college or AA degree	590 (31.6%)	492 (29.4%)	
College graduate or above	445 (23.8%)	428 (25.6%)	
Marital status (*n*, %)			<0.001
Married/Living with Partner	1,182 (63.2%)	939 (56.1%)	
Widowed/Divorced/Separated	519 (27.8%)	616 (36.8%)	
Never married	169 (9.04%)	120 (7.16%)	
Ratio of family income to poverty (*n*, %)			0.011
≤1	298 (15.9%)	313 (18.7%)	
1 ~ 3	791 (42.3%)	738 (44.1%)	
>3	781 (41.8%)	624 (37.3%)	
Smoke (*n*, %)			<0.001
Yes	927 (49.6%)	723 (43.2%)	
No	943 (50.4%)	952 (56.8%)	
Drinking alcohol (*n*, %)			<0.001
Yes	368 (19.7%)	240 (14.3%)	
No	1,502 (80.3%)	1,435 (85.7%)	
BMI (*n*, %)			<0.001
<25	303 (16.2%)	580 (34.6%)	
25 ~ 30	680 (36.4%)	647 (38.6%)	
≥30	887 (47.4%)	448 (26.7%)	
Diabetes (*n*, %)			<0.001
Yes	489 (26.1%)	310 (18.5%)	
No	1,381 (73.9%)	1,365 (81.5%)	
Hypertension (*n*, %)			0.319
Yes	1,017 (54.4%)	882 (52.7%)	
No	853 (45.6%)	793 (47.3%)	
History of personal osteoporosis and fracture (*n*, %)			<0.001
Yes	560 (29.9%)	612 (36.5%)	
No	1,310 (70.1%)	1,063 (63.5%)	
Parental history of osteoporosis and fracture (*n*, %)			<0.001
Yes	315 (16.8%)	410 (24.5%)	
No	1,555 (83.2%)	1,265 (75.5%)	
Direct HDL-Cholesterol (mmol/L)	1.36 (0.40)	1.50 (0.45)	<0.001
Total Cholesterol (mmol/L)	4.84 (1.13)	5.01 (1.14)	<0.001
White blood cell count (1000 cells/uL)	7.21 (9.31)	6.91 (2.20)	0.181
Lymphocyte percent (%)	30.9 (9.27)	30.0 (9.10)	0.003
Monocyte percent (%)	8.60 (2.59)	8.37 (2.12)	0.003
Segmented neutrophils percent (%)	56.8 (9.92)	58.1 (9.62)	<0.001
Eosinophils percent (%)	2.96 (2.22)	2.84 (2.04)	0.079
Basophils percent (%)	0.83 (0.34)	0.84 (0.35)	0.464
Lymphocyte number (1,000 cells/uL)	2.35 (8.55)	2.12 (3.41)	0.303
Monocyte number (1,000 cells/uL)	0.59 (0.25)	0.56 (0.20)	0.001
Segmented neutrophils num (1,000 cell/uL)	4.08 (1.74)	4.08 (1.63)	0.948
Eosinophils number (1,000 cells/uL)	0.21 (0.18)	0.19 (0.16)	0.013
Basophils number (1,000 cells/uL)	0.05 (0.05)	0.05 (0.05)	0.696
Red blood cell count (million cells/uL)	4.76 (0.50)	4.59 (0.49)	<0.001
Hemoglobin (g/dL)	14.2 (1.53)	13.9 (1.41)	<0.001
Hematocrit (%)	42.2 (4.16)	41.3 (3.92)	<0.001
Mean cell volume (fL)	89.0 (6.06)	90.1 (5.51)	<0.001
Mean cell hemoglobin concentration (g/dL)	33.6 (0.95)	33.5 (0.85)	0.229
Mean cell hemoglobin (pg)	29.9 (2.47)	30.2 (2.18)	<0.001
Red cell distribution width (%)	14.0 (1.26)	13.9 (1.27)	0.003
Platelet count (1,000 cells/uL)	230 (61.4)	236 (65.5)	0.006
Mean platelet volume (fL)	8.32 (0.93)	8.24 (0.91)	0.012
Nucleated red blood cells	0.09 (0.09)	0.08 (0.08)	0.036
Glycohemoglobin (%)	6.22 (1.26)	6.00 (1.13)	<0.001
Alanine aminotransferase (ALT) (U/L)	22.9 (16.3)	20.4 (20.8)	<0.001
Albumin, refrigerated serum (g/L)	40.5 (3.22)	40.3 (3.27)	0.321
Alkaline phosphatase (ALP) (IU/L)	79.6 (26.1)	83.8 (27.3)	<0.001
Aspartate aminotransferase (AST) (U/L)	22.5 (12.9)	22.0 (15.7)	0.346
Bicarbonate (mmol/L)	25.6 (2.47)	25.8 (2.52)	0.028
Blood urea nitrogen (mmol/L)	5.91 (2.26)	5.94 (2.27)	0.741
Chloride (mmol/L)	101 (3.06)	101 (3.21)	0.045
Creatine phosphokinase (CPK) (IU/L)	175 (232)	117 (116)	<0.001
Creatinine, refrigerated serum (umol/L)	85.5 (43.4)	79.3 (41.4)	<0.001
Globulin (g/L)	31.0 (4.43)	30.4 (4.58)	<0.001
Glucose, refrigerated serum (mmol/L)	6.13 (2.51)	5.84 (2.15)	<0.001
Gamma glutamyl transferase (GGT) (IU/L)	37.5 (46.9)	30.5 (40.4)	<0.001
Iron, refrigerated serum (umol/L)	15.9 (6.12)	16.1 (6.16)	0.384
Lactate dehydrogenase (LDH) (IU/L)	163 (36.3)	167 (36.1)	0.009
Osmolality (mmol/Kg)	283 (5.42)	282 (6.08)	0.069
Phosphorus (mmol/L)	1.14 (0.18)	1.15 (0.16)	0.011
Potassium (mmol/L)	4.12 (0.40)	4.13 (0.39)	0.660
Sodium (mmol/L)	141 (2.63)	141 (2.98)	0.597
Total bilirubin (umol/L)	8.44 (5.00)	7.99 (4.93)	0.007
Total calcium (mmol/L)	2.32 (0.09)	2.33 (0.10)	0.017
Total protein (g/L)	71.5 (4.37)	70.8 (4.65)	<0.001
Uric acid (umol/L)	344 (88.6)	312 (85.7)	<0.001

BMI, body mass index.

### 3.2 Feature selection

**Figure 2:**
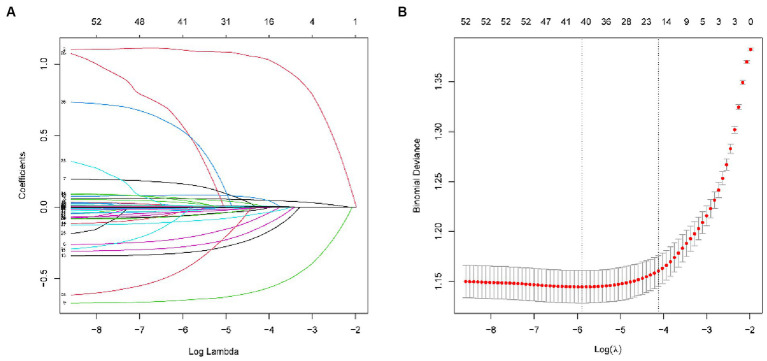
**(A)** Lasso coefficient path plots for 59 variables. **(B)** Cross-validation curves (10-fold cross validation). The left dashed line represents lambda.min and the right dashed line represents lambda.1se.

Variable selection was performed by Lasso (Least Absolute Shrinkage and Selection Operator), as shown in Figure 2, and 10-fold cross-validation was used to select λ. Due to the large number of characteristic variables in this study, if λ.min is used as the optimal λ value, there will be 41 variables included in the final model, which makes the model too complex and may have the risk of overfitting. On the other hand, when λ.1se is chosen as the optimal λ value, 19 variables will be included in the model, which is more concise and has a good prediction performance. Therefore, λ.1se is finally chosen as the optimal λ value in this study. The 19 variables included in the machine learning model were Age, Gender, Ratio of family income to poverty, BMI, Diabetes, and History of personal osteoporosis and fracture, Parental history of osteoporosis and fracture, Total Cholesterol, Monocyte percent, Segmented neutrophils percent, Mean cell volume, Red cell distribution width, Glycohemoglobin, Alkaline Phosphatase (ALP), Creatine Phosphokinase (CPK), Globulin, Osmolality, Total Protein, Uric acid.

### 3.3 Evaluation of model performance

**Figure 3:**
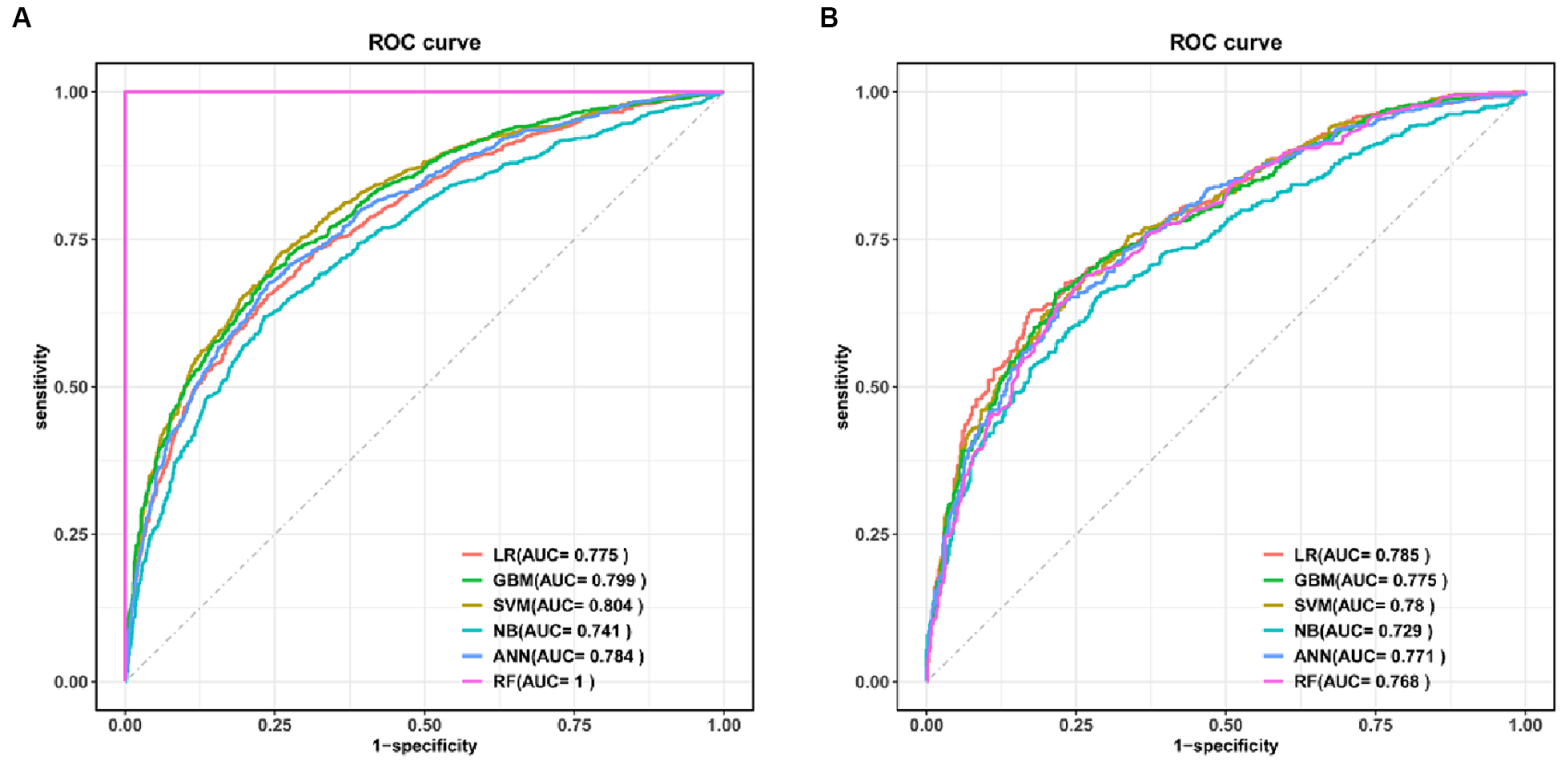
ROC curves for the six models in the training set **(A)** and test set **(B)**. LR, logistic regression; SVM, support vector machine; GBM, gradient boosting machine; NB, naive Bayesian; ANN, artificial neural network; RF, random forest.

**Figure 4:**
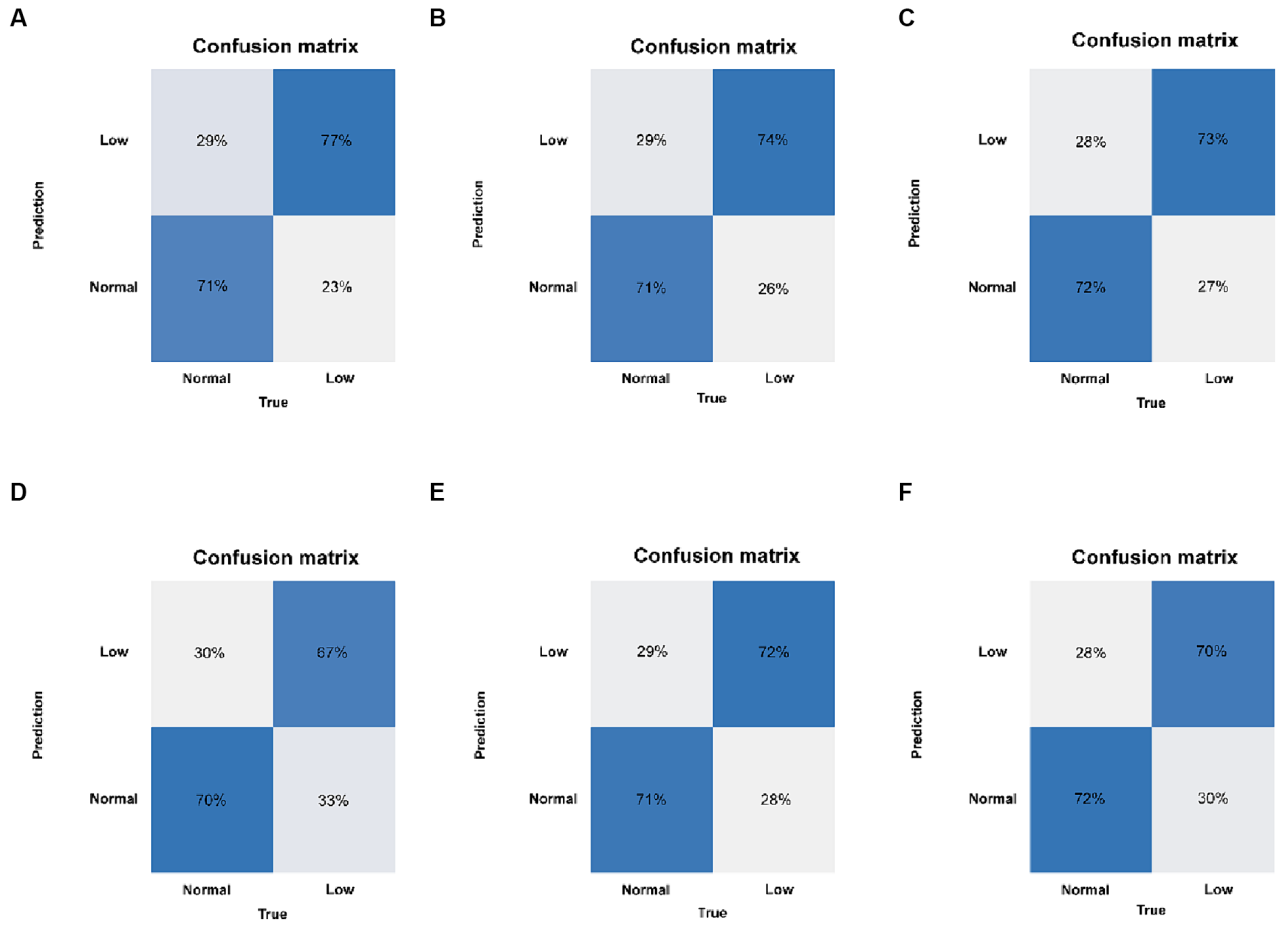
Confusion matrix of the six models in the test set. **(A)** LR, logistic regression. **(B)** SVM, support vector machine. **(C)** GBM, gradient boosting machine. **(D)** NB, naive Bayesian. **(E)** ANN, artificial neural network. **(F)** RF, random forest.

**Figure 5:**
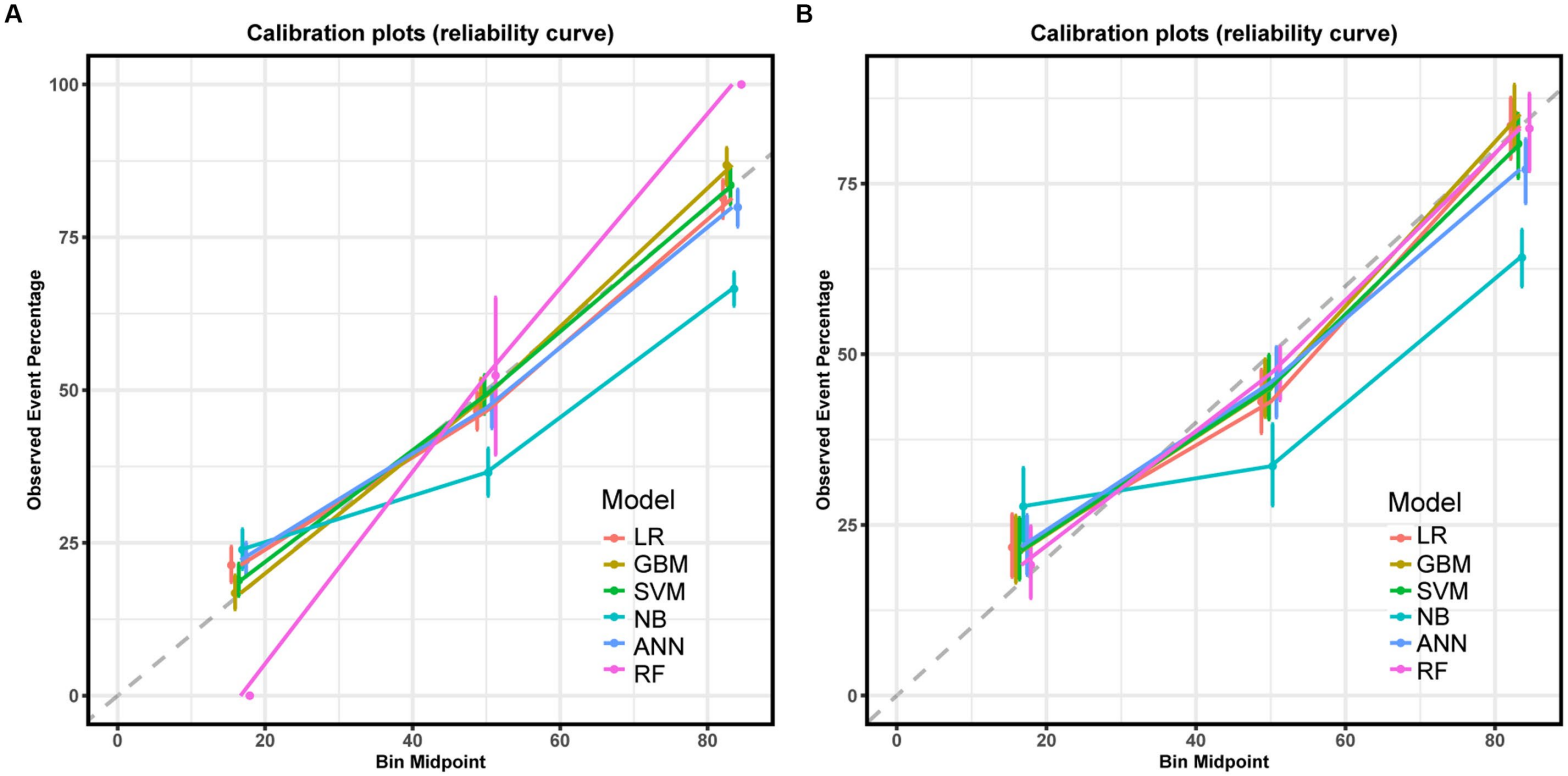
Calibration curve for the six models in the training set **(A)** and test set **(B)**.

**Figure 6:**
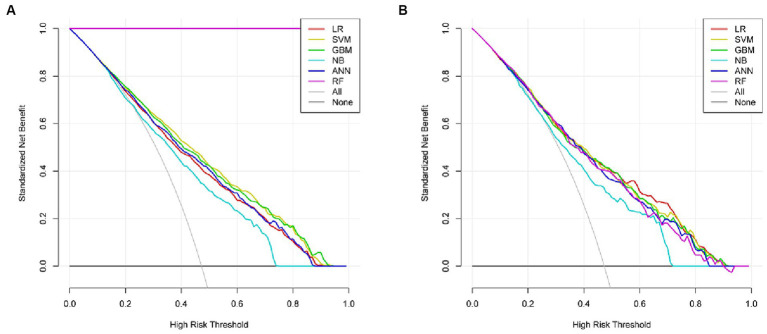
Decision curves for the six models in the training set **(A)** and test set **(B)**.

Six machine learning models were constructed in this study, Figure 3 shows the ROC curves for the training and test sets of the model in the internal validation set, in the test set, LR (AUC = 0.785) has the highest AUC value and the best model discrimination, followed by SVM (AUC = 0.78), GBM (AUC = 0.775), ANN (AUC = 0.771), RF (AUC = 0.761), and NB (AUC = 0.729); LR also had higher accuracy (0.733), specificity (0.829), and precision (0.766) than the remaining five models; RF had the highest sensitivity (0.684); and GBM had a higher F1 score (0.693) than the other models (Table 2). Figure 4 shows the confusion matrix for the model test set, from which it can also be seen that LR has the strongest ability to discriminate between people with normal bone density and those with low bone density among the six models. The calibration curves of the six model training and validation sets are shown in Figure 5, and in the test set, the calibration curve of RF fits the ideal curve to the highest degree, and the calibration curves of the rest of the models fit the ideal curve reasonably well except for NB, which has a worse fit, suggesting a better match between the predicted probabilities of the models and the actual observed incidence rates. The results of Decision Curve Analysis (DCA) on the training and test sets of the models are shown in Figure 6, which shows that when the predictive probability threshold is certain, LR has the largest net gain compared to the other five models, indicating that LR has better clinical utility. In the external validation of the model, the AUC value (0.78), accuracy (0.718), specificity (0.752), and precision (0.667) of LR were higher than those of the other models, and good robustness and extrapolation ability could also be seen from the confusion matrix, ROC curve, calibration curve, and decision curve of the model (Supplementary Figures S3, S4 and Supplementary Table S3). Therefore, from the comprehensive evaluation of model differentiation, calibration, and clinical gain, LR is the optimal model for predicting low BMD population.

**Table 2:** Comparison of the predictive power of several models in the test set.

Model	AUC	Accuracy	Sensitivity	Specificity	Precision	F1
LR	0.785	0.733	0.626	0.829	0.766	0.689
SVM	0.78	0.718	0.628	0.799	0.737	0.678
GBM	0.775	0.725	0.658	0.784	0.732	0.693
NB	0.729	0.685	0.66	0.708	0.669	0.665
ANN	0.771	0.712	0.642	0.775	0.719	0.679
RF	0.768	0.712	0.684	0.738	0.701	0.692

### 3.4 Evaluation of the importance of variables

**Figure 7:**
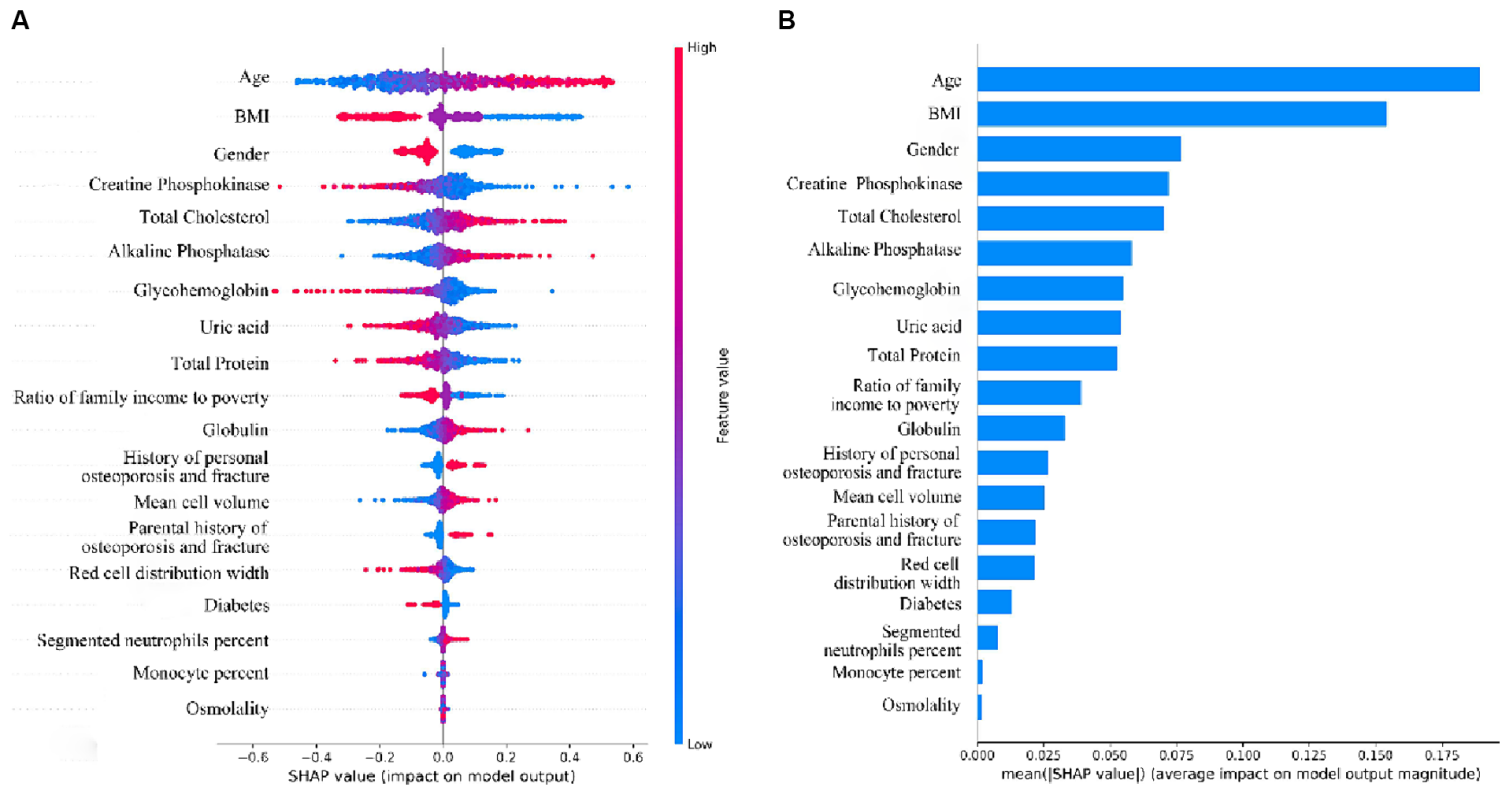
**(A)** Beeswarm plots of the LR Model. Generate SHAP values for each variable and reveal its relationship with low bone density. **(B)** Importance ranking plot of variables for LR model.

We interpreted the importance of predictor variables based on the SHAP algorithm for the LR model with the best predictive performance (Figure 7). The extent to which a variable contributes to the model is reflected by the SHAP value. A higher SHAP value of a variable means a higher degree of its contribution to the model (26). As shown in Figure 7A, the top-down ordering of the variables means that their contribution to low BMD is in ascending order, with the line with a SHAP value of 0 as the vertical axis, the variables with red color on the right side of the line represent the positive contribution of the variable to the predicted outcome, while the variables with blue color on the right side of the line have a negative contribution. Therefore, the top six variables in terms of importance for predicting low bone mass in the population were: age > BMI > gender > creatine phosphokinase > total cholesterol > alkaline phosphatase, in which age, total cholesterol, and alkaline phosphatase were positively correlated with the occurrence of low bone mineral density, i.e., the older the age, the higher the indexes of total cholesterol and alkaline phosphatase, and the higher the probability of developing low bone mineral density. BMI, gender, and creatine phosphokinase were negatively correlated with the occurrence of low BMD, i.e., the lower the BMI, the female, and the lower the creatine phosphokinase index, the higher the probability of low BMD. Given that age was the variable with the highest variable importance in the model of this study, we explored the effect of age on the occurrence of low BMD as well as other blood biochemical indices. Comparison of the study subjects divided into groups with a cutoff of 5 years of age revealed that most of the blood biochemical indices were significantly associated with age (Supplementary Table S2). Their associations were further explored by applying restricted cubic spline (RCS), and age was found to be linearly related to the occurrence of low BMD, with the older the age, the higher the risk of low BMD (Supplementary Figure S1). Among the blood biochemical indices, except for Alkaline Phosphatase (ALP), Mean cell volume, Segmented neutrophils percent, and Total Cholesterol, all of them showed a linear trend with age (Supplementary Figure S2).

## 4 Discussion

With the aging of the population worldwide in recent years, the incidence of osteoporosis in older adult/adults men and women remains high, and fractures caused by osteoporosis can lead to disability, prolonged bed rest, impaired function, and even death, bringing serious economic and physical and psychological burdens to the affected families as well as to individuals (27). Some studies have shown that early diagnosis and intervention for patients with osteopenia and osteoporosis can effectively reduce their fracture incidence (28), so we developed several machine learning algorithms to identify abnormal bone density in the population with osteopenia and osteoporosis. In medical research, the collection of clinical data is difficult and the collected data are heterogeneous and non-standardized, while public databases such as SEER, MIMIC, and NHANES have the advantages of large amount of data and richness of the information contained in them, and thus they are widely favored by researchers (29). Many previous studies (30–32) have applied machine learning algorithms to mine public databases and achieved good prediction results. Our study included 3,545 participants with complete femoral neck BMD measurements from 2017 to 2020 from the National Household Nutrition and Exercise Survey (NHANES) database, which were divided into a training set and a test set according to the ratio of 7:3, with 2,841 participants in the training set and 1,064 participants in the test set, and the data from the training set were analyzed by using demographic factors, blood biochemical indices, and questionnaire information, which are clinically readily available variables, six common supervised machine learning models were built using the training set data and the model performance was tested with the test set data, and the model with the best predictive performance, LR, was finally selected based on the ROC curves, calibration curves, decision curves, confusion matrices, as well as model performance evaluation indexes, such as accuracy and sensitivity, etc. It is worth noting that the performances of the three models, GBM, SVM, and ANN, are also very well. Especially in the training set (Table 3), the AUC values of SVM (AUC = 0.804), GBM (AUC = 0.799), and ANN (AUC = 0.784) even exceed that of LR (AUC = 0.775), and it can be seen from the calibration curves and the decision curves of the training set that the fit of the calibration curves of GBM and SVM is better than that of LR, and ANN is on a par with LR. The decision curve performance of GBM, SVM and ANN is also better than that of LR. The ability of two models, RF and NB, to predict the population with low bone density is relatively weak. RF has an overfitting problem in the training set, and in the test set, although the calibration curves fit the ideal curves better, the AUC value is low, and the model’s differentiation is average. Several model evaluation indexes of NB are lower in the training set and the test set. The model’s ROC curve, calibration curve, and decision curve are poor compared to the rest of the models, and the predictive ability is the weakest among the six models.

**Table 3:** Comparison of the predictive ability of several models in the training set.

Model	AUC	Accuracy	Sensitivity	Specificity	Precision	F1
LR	0.775	0.712	0.657	0.761	0.711	0.683
SVM	0.804	0.734	0.728	0.739	0.714	0.721
GBM	0.799	0.728	0.687	0.765	0.724	0.705
NB	0.741	0.697	0.619	0.767	0.704	0.659
ANN	0.784	0.721	0.677	0.76	0.716	0.696
RF	1	1	1	1	1	1

We analyzed the variable importance of the 19 independent variables included in the model through the SHAP framework, and found that the top three variables in terms of importance were age, BMI, and gender, and that older age, lower BMI, and female gender were risk factors for lower BMD. In previous studies, age and gender have been recognized as established risk factors for osteoporosis (33, 34), especially in women, after menopause, the level of estrogen in the body decreases, and BMD decreases, and the prevalence of osteoporosis rises dramatically, so that women over the age of 50 years are often a priority population for osteoporosis screening (35). Whereas the relationship between BMI and BMD is unclear, a two-sample Mendelian randomization study showed a positive causal association between BMI and BMD levels (36); a meta-analysis that included 108 studies showed that the risk of osteoporosis in people with low BMI was 2.76 times higher than that in people with high BMI (6), which are in keeping with the conclusions we have drawn. However, a prospective study concluded that the contribution of BMI to fragility fractures varies by gender and by skeletal site, with a more complex association between the two (37). Therefore, further exploration of the relationship between BMI and BMD is warranted.

Among the blood biochemical indices, the three variables that contribute most to low BMD are creatine phosphokinase, total cholesterol, and alkaline phosphatase, where the higher the two indices of total cholesterol and alkaline phosphatase, the higher the likelihood of lower BMD, and the opposite is true of creatine phosphokinase, where the lower the value, the higher the likelihood of lower BMD. Creatine phosphokinase (CPK), also known as creatine kinase (CK), plays an important role in cellular energy metabolism, and fewer studies have been conducted on the association between CK and BMD. A retrospective and prospective cohort study found that the group with a history of previous fracture had a higher level of CK values than the group without a history of fracture, and the group that presented with a new fracture also had a higher level than the group that did not present with a fracture, which is contrary to our opinion, but the study was only conducted on young female athletes, which has some limitations, and the number of subjects was small, so this conclusion also needs to be further confirmed (38). Alkaline phosphatase is a bone turnover marker that is widely found in bone, liver, and intestine and plays an important role in bone growth and metabolism (39). Previous studies have shown that higher ALP levels are positively associated with low BMD or osteoporosis, which is consistent with the conclusions we have drawn, probably because alkaline phosphatase activity is increased when skeletal disease is present to meet the demands of bone growth and reconstruction (40, 41). There is no clear consensus on the relationship between total cholesterol and BMD, and most studies agree with us (42–44) that there is a negative correlation between the two, however, there are also studies that take the opposite view (40), and a cross-sectional study from China found that the associations were very different in men and women, with TC positively correlated with BMD in men and In women, the association was U-shaped, with curve inflection points varying by age and BMI (45). Therefore, the association and mechanisms between TC and BMD need to be explored in further studies.

The present study also has some limitations. First, in the NHANES database, those who participated in BMD measurement by dual-energy X-ray absorptiometry were older than 50 years, and nowadays there is a trend of younger age for both osteoporosis and bone loss (46), so screening should not be limited to the middle-aged and older population. Second, our study is based on the U.S. NHANES database, which, although covering multiple races in the U.S., may have limitations when applied to other racial or national populations. Therefore, data from different countries and regions will be collected and analyzed in the future to increase the generalizability of the model. Third, although several variables such as demographic and blood biochemical indicators were included in this study, there are many factors that were not included in the study, such as lifestyle, dietary habits, genomic data, and imaging data, which are also closely related to BMD. It is hoped that more data such as these will be included in future studies to further improve the accuracy of the model and expand its scope of application. Fourth, with the rapid development of the field of artificial intelligence, new algorithms such as deep learning algorithms (47, 48) and image recognition technology (49) are constantly emerging. In addition, more and more research tends to explore diseases from the perspective of pathogenic mechanisms (50) and drug development (51), and we are looking forward to making more progress in these areas in the future.

## 5 Conclusion

In this study, we applied six machine learning algorithms to construct a prediction model for low bone mass based on clinically accessible metrics in the NHANES database, and used 10-fold cross-validation to internally validate the model and NHANES data from different time periods to input into the model as an external validation, applying multiple metrics to evaluate the model performance, and finally selecting the best predictive performance of the ML model, LR. The model can screen out people osteopenia and osteoporosis, and assist clinicians in making decisions to better realize the primary and secondary prevention of osteoporosis.

## Data availability statement

The original contributions presented in the study are included in the article/Supplementary material, further inquiries can be directed to the corresponding authors.

## Ethics statement

The NHANES protocols and studies involving human participants were reviewed and approved by NCHS Research Ethics Review Board. The patients/participants provided their written informed consent to participate in this study.

## Author contributions

RX: Conceptualization, Data curation, Methodology, Software, Writing – original draft. YC: Data curation, Software, Writing – review & editing. ZY: Methodology, Writing – review & editing. WW: Data curation, Writing – review & editing. JC: Validation, Writing – review & editing. RW: Software, Writing – review & editing. YD: Methodology, Writing – review & editing. CJ: Data curation, Writing – review & editing. ZH: Supervision, Writing – review & editing. XL: Supervision, Writing – review & editing.
