# Supplementary material for: Application of machine learning algorithms to identify people with low bone density
Source: Front Public Health. 2024 Apr 25;12:1347219. doi: 10.3389/fpubh.2024.1347219 (PMC11080984; doi:10.3389/fpubh.2024.1347219)
Supplement: Supplementary file 1 [file Data_Sheet_1.docx]

**

**

**Supplemental Figure1|**Restricted cubic spline(RCS) plots of the relationship between age and the occurrence of low bone density.


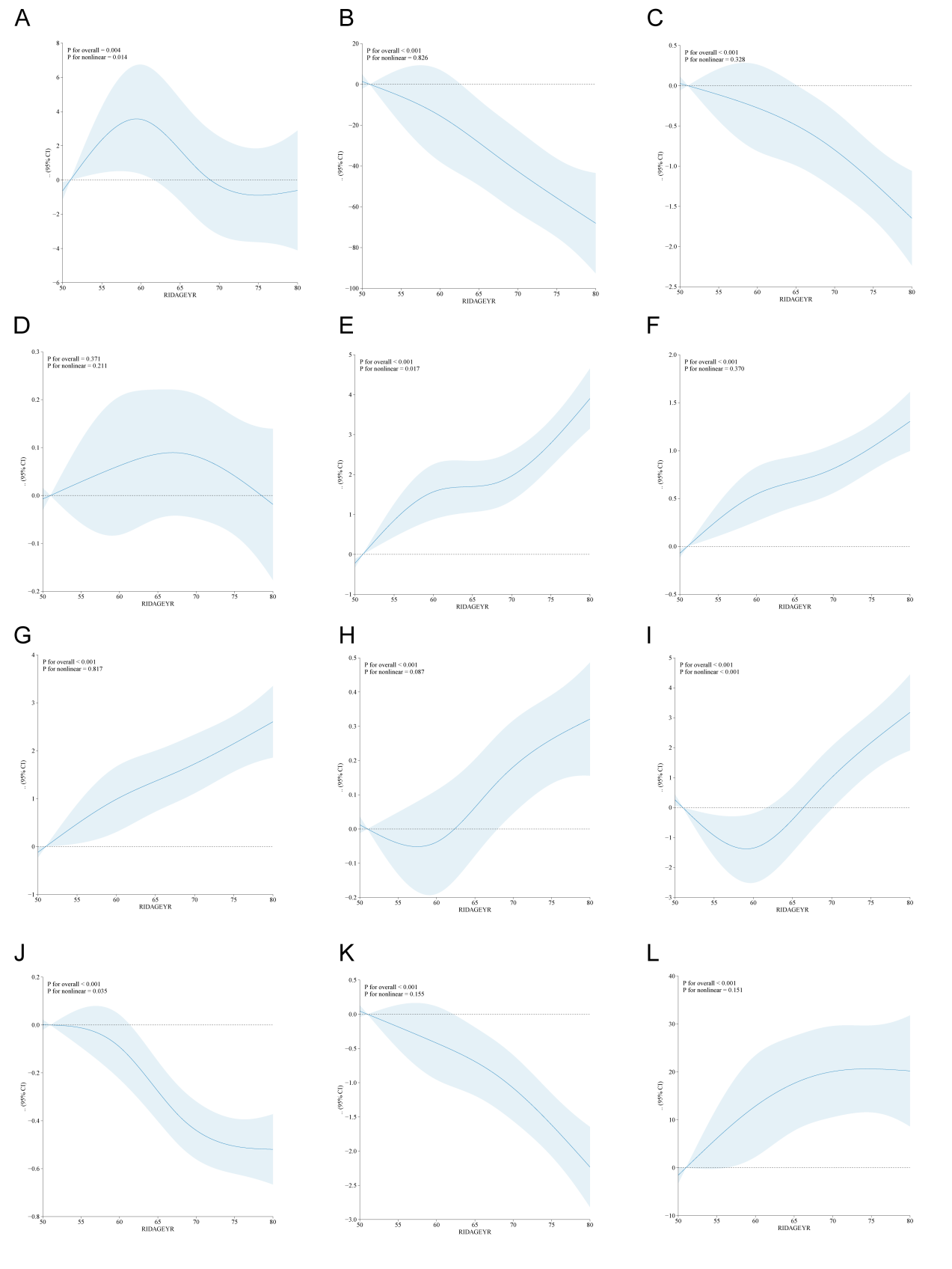


**Supplemental Figure2|**Restricted cubic spline(RCS) plots of the relationship between age and various blood biochemicals. (A)Alkaline Phosphatase (ALP). (B)Creatine Phosphokinase (CPK). (C)Globulin. (D)Glycohemoglobin. (E)Mean cell volume. (F)Monocyte percent. (G)Osmolality. (H)Red cell distribution width. (I)Segmented neutrophils percent. (J)Total Cholesterol. (K)Total Protein. (L)Uric acid.

**
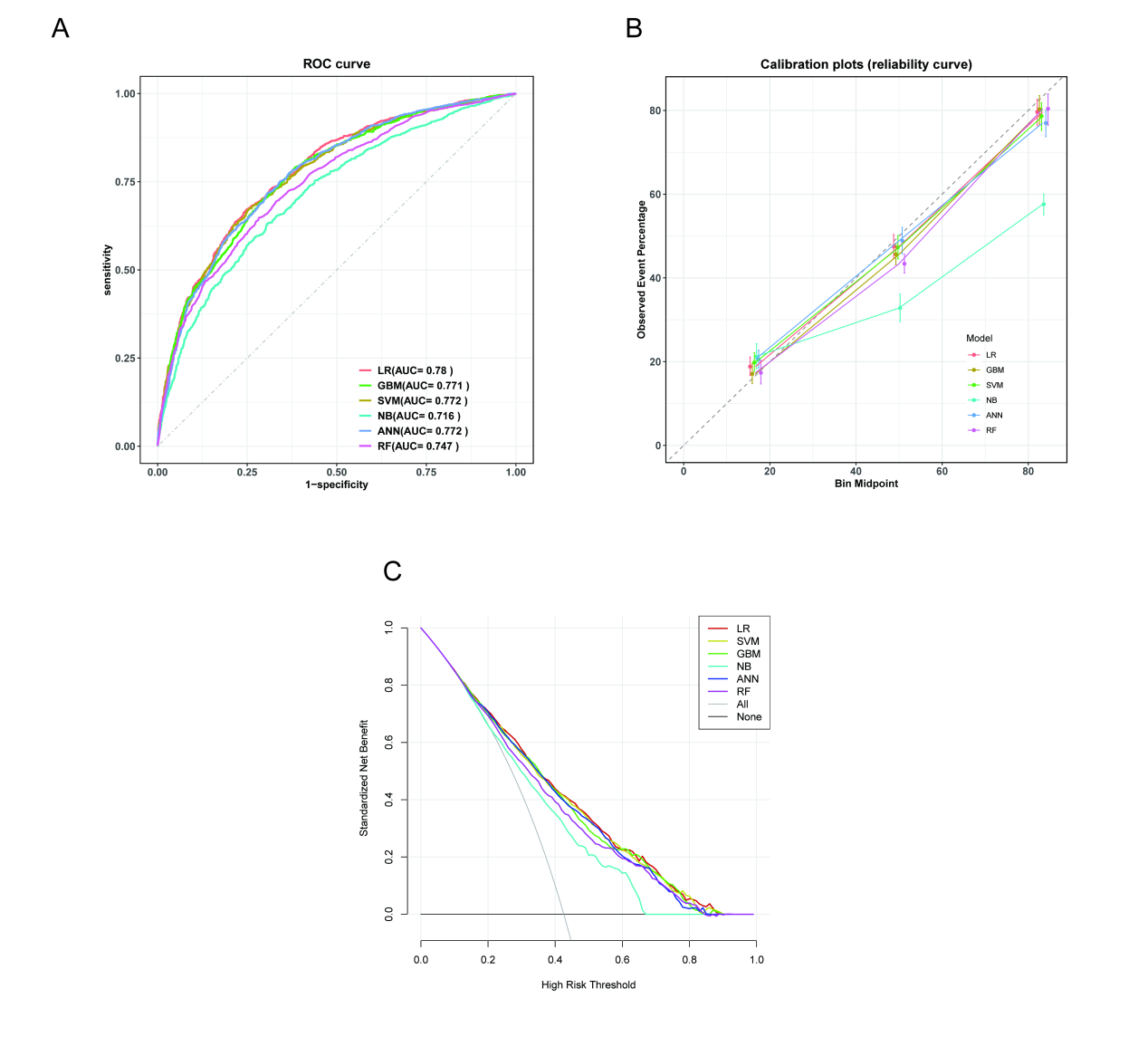
**

**Supplemental Figure3|**(A)ROC curves for external validation of six models. (B)calibration curves for external validation of six models. (C)Decision curves for external validation of six models.


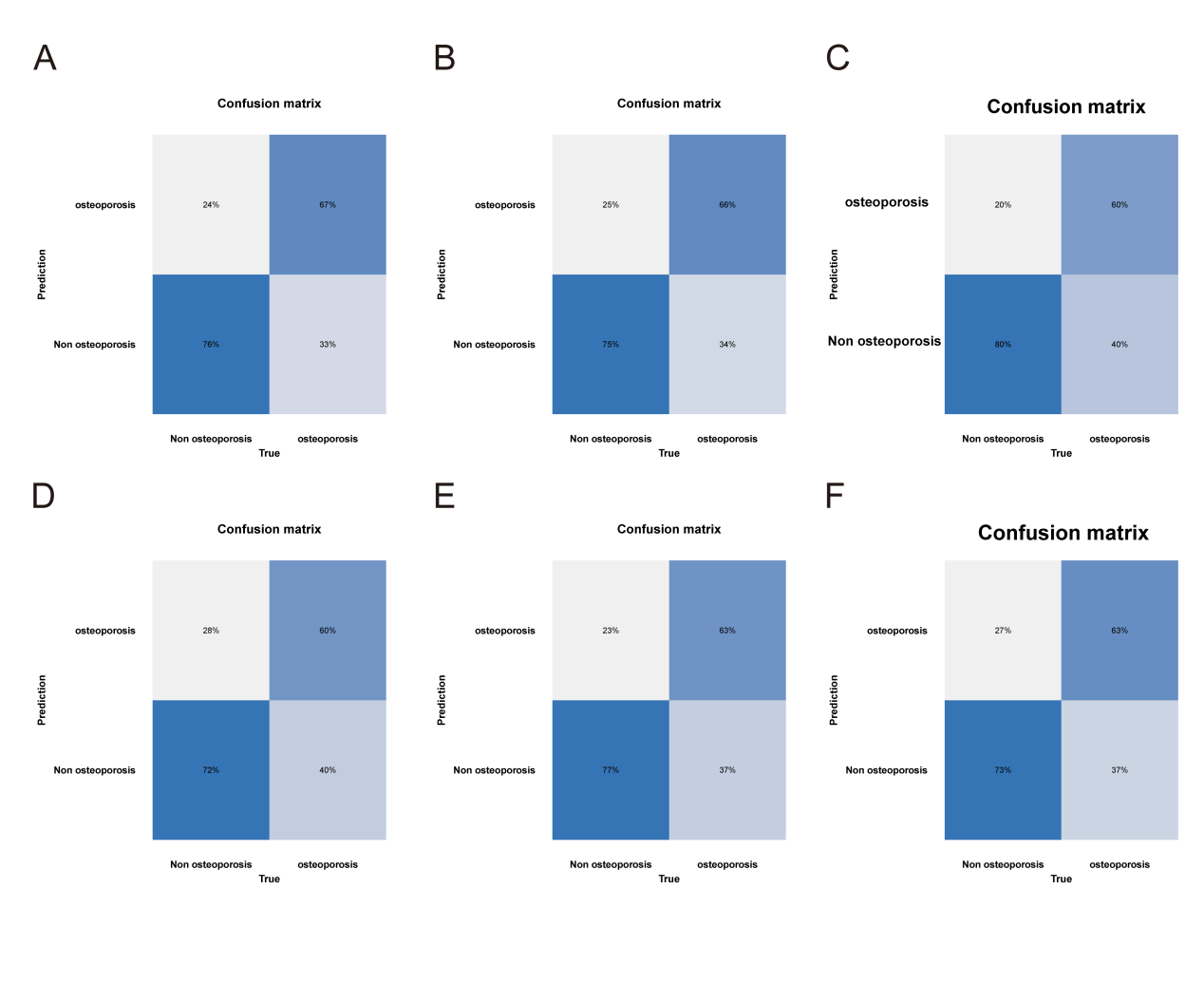


**Supplemental Figure4|**Confusion matrix for six models in external validation. (A)LR,logistic regression. (B)SVM,support vector machine. (C)GBM,gradient boosting machine. (D)NB,naive bayesian. (E)ANN,artificial neural network. (F)RF,random forest.
