# Supplementary material for: Application of machine learning algorithms to identify people with low bone density
Source: Front Public Health. 2024 Apr 25;12:1347219. doi: 10.3389/fpubh.2024.1347219 (PMC11080984; doi:10.3389/fpubh.2024.1347219)
Supplement: Supplementary file 2 [file Data_Sheet_2.docx]

**Supplemental Table1|**Comparison of baseline data between study participants in the low and normal bone density groups in the external validation set.

|  | **Normal bone density** | **Low bone density** | *P* |
| --- | --- | --- | --- |
|  | ***N=1796*** | ***N=1331*** |  |
| Age(year) | 55.2 (11.1) | 62.9 (11.6) | <0.001 |
| Gender(n,%) |  |  | <0.001 |
| Male | 1075 (59.9%) | 471 (35.4%) |  |
| Female | 721 (40.1%) | 860 (64.6%) |  |
| Ratio of family income to poverty(n,%) |  |  | 0.164 |
| ≤1 | 330 (18.4%) | 260 (19.5%) |  |
| 1~3 | 664 (37.0%) | 522 (39.2%) |  |
| >3 | 802 (44.7%) | 549 (41.2%) |  |
| BMI(n,%) |  |  | <0.001 |
| <25 | 346 (19.3%) | 538 (40.4%) |  |
| 25~30 | 658 (36.6%) | 463 (34.8%) |  |
| ≥30 | 792 (44.1%) | 330 (24.8%) |  |
| Diabetes(n,%) |  |  | 0.243 |
| Yes | 303 (16.9%) | 203 (15.3%) |  |
| No | 1493 (83.1%) | 1128 (84.7%) |  |
| History of personal osteoporosis and fracture(n,%) |  |  | <0.001 |
| Yes | 483 (26.9%) | 461 (34.6%) |  |
| No | 1313 (73.1%) | 870 (65.4%) |  |
| Parental history of osteoporosis and fracture(n,%) |  |  | <0.001 |
| Yes | 259 (14.4%) | 299 (22.5%) |  |
| No | 1537 (85.6%) | 1032 (77.5%) |  |
| Total Cholesterol(mmol/L) | 5.04 (1.16) | 5.08 (1.07) | 0.300 |
| Monocyte percent (%) | 57.6 (9.47) | 58.3 (9.36) | 0.037 |
| Segmented neutrophils percent (%) | 8.36 (2.28) | 8.31 (2.23) | 0.532 |
| Mean cell volume (fL) | 89.0 (6.10) | 90.4 (6.00) | <0.001 |
| Red cell distribution width (%) | 13.8 (1.27) | 13.7 (1.17) | 0.275 |
| Glycohemoglobin (%) | 5.95 (1.26) | 5.85 (0.99) | 0.009 |
| Alkaline Phosphatase (ALP) (IU/L) | 66.0 (22.5) | 69.4 (26.5) | <0.001 |
| Creatine Phosphokinase (CPK) (IU/L) | 162 (175) | 116 (107) | <0.001 |
| Globulin (g/L) | 28.5 (4.55) | 28.0 (4.75) | 0.007 |
| Osmolality (mmol/Kg) | 280 (5.38) | 281 (5.82) | <0.001 |
| Total Protein (g/L) | 70.6 (4.66) | 70.1 (5.15) | 0.005 |
| Uric acid (umol/L) | 334 (84.6) | 313 (82.2) | <0.001 |

Abbreviations:BMI,body mass index.

**Supplemental Table2|**Comparison of Baseline Characteristics of Study Participants Based on Different Age Groups.

|  |  | 50-54 | 55-59 | 60-64 | 65-69 | 70-74 | 75-80 | p |
| --- | --- | --- | --- | --- | --- | --- | --- | --- |
|  | Level | n=540 | n=622 | n=785 | n=537 | n=417 | n=644 |  |
| Gender (%) | Male | 279 (51.7) | 294 (47.3) | 427 (54.4) | 288 (53.6) | 230 (55.2) | 358 (55.6) | 0.038 |
|  | Female | 261 (48.3) | 328 (52.7) | 358 (45.6) | 249 (46.4) | 187 (44.8) | 286 (44.4) |  |
| Ratio of family income to poverty (%) | ≤1 | 107 (19.8) | 111 (17.8) | 165 (21.0) | 101 (18.8) | 54 (12.9) | 73 (11.3) | <0.001 |
|  | 1~3 | 208 (38.5) | 228 (36.7) | 313 (39.9) | 247 (46.0) | 194 (46.5) | 339 (52.6) |  |
|  | >3 | 225 (41.7) | 283 (45.5) | 307 (39.1) | 189 (35.2) | 169 (40.5) | 232 (36.0) |  |
| BMI (%) | <25 | 107 (19.8) | 162 (26.0) | 177 (22.5) | 127 (23.6) | 99 (23.7) | 211 (32.8) | <0.001 |
|  | 25~30 | 208 (38.5) | 220 (35.4) | 299 (38.1) | 188 (35.0) | 156 (37.4) | 256 (39.8) |  |
|  | ≥30 | 225 (41.7) | 240 (38.6) | 309 (39.4) | 222 (41.3) | 162 (38.8) | 177 (27.5) |  |
| Diabetes (%) | Yes | 92 (17.0) | 101 (16.2) | 181 (23.1) | 148 (27.6) | 107 (25.7) | 170 (26.4) | <0.001 |
|  | No | 448 (83.0) | 521 (83.8) | 604 (76.9) | 389 (72.4) | 310 (74.3) | 474 (73.6) |  |
| History of personal osteoporosis and fracture (%) | Yes | 134 (24.8) | 213 (34.2) | 237 (30.2) | 195 (36.3) | 155 (37.2) | 238 (37.0) | <0.001 |
|  | No | 406 (75.2) | 409 (65.8) | 548 (69.8) | 342 (63.7) | 262 (62.8) | 406 (63.0) |  |
| Parental history of osteoporosis and fracture (%) | Yes | 103 (19.1) | 132 (21.2) | 164 (20.9) | 122 (22.7) | 93 (22.3) | 111 (17.2) | 0.179 |
|  | No | 437 (80.9) | 490 (78.8) | 621 (79.1) | 415 (77.3) | 324 (77.7) | 533 (82.8) |  |
| Total Cholesterol (mean (SD)) |  | 5.13 (1.07) | 5.17 (1.10) | 4.99 (1.15) | 4.81 (1.13) | 4.71 (1.12) | 4.65 (1.14) | <0.001 |
| Monocyte percent (mean (SD)) |  | 7.97 (2.00) | 8.16 (2.17) | 8.50 (2.25) | 8.55 (2.40) | 8.75 (2.15) | 9.04 (2.96) | <0.001 |
| Segmented neutrophils percent (mean (SD)) |  | 56.95 (9.44) | 56.20 (9.59) | 55.76 (9.75) | 57.70 (9.65) | 58.73 (9.93) | 59.97 (9.76) | <0.001 |
| Mean cell volume (mean (SD)) |  | 88.00 (6.17) | 89.14 (5.62) | 89.17 (5.78) | 89.33 (5.58) | 90.38 (5.79) | 91.21 (5.59) | <0.001 |
| Red cell distribution width (mean (SD)) |  | 13.88 (1.32) | 13.75 (1.17) | 13.90 (1.12) | 14.04 (1.47) | 14.08 (1.19) | 14.15 (1.32) | <0.001 |
| Glycohemoglobin (mean (SD)) |  | 6.08 (1.35) | 6.09 (1.31) | 6.16 (1.33) | 6.15 (1.06) | 6.11 (1.02) | 6.10 (1.00) | 0.819 |
| Alkaline Phosphatase (ALP) (mean (SD)) |  | 81.41 (26.95) | 83.80 (26.41) | 82.67 (27.43) | 81.41 (27.57) | 80.14 (23.83) | 79.43 (27.04) | 0.051 |
| Creatine Phosphokinase (CPK) (mean (SD)) |  | 169.76 (151.38) | 168.97 (345.73) | 153.32 (142.27) | 148.46 (137.86) | 127.44 (114.32) | 113.10 (103.14) | <0.001 |
| Globulin (mean (SD)) |  | 31.48 (4.10) | 30.85 (4.58) | 31.03 (4.47) | 31.11 (4.85) | 29.90 (4.20) | 29.96 (4.57) | <0.001 |
| Osmolality (mean (SD)) |  | 281.26 (5.53) | 281.91 (5.33) | 282.17 (5.55) | 282.73 (5.40) | 282.95 (6.20) | 283.64 (6.22) | <0.001 |
| Total Protein (mean (SD)) |  | 72.01 (4.27) | 71.64 (4.51) | 71.47 (4.36) | 71.40 (4.79) | 70.39 (4.21) | 70.14 (4.64) | <0.001 |
| Uric acid (mean (SD)) |  | 318.45 (91.85) | 320.20 (86.59) | 329.45 (86.95) | 338.41 (91.93) | 330.84 (83.18) | 335.87 (89.05) | <0.001 |
| Bone (%) | No | 361 (66.9) | 383 (61.6) | 455 (58.0) | 276 (51.4) | 167 (40.0) | 228 (35.4) | <0.001 |
|  | Yes | 179 (33.1) | 239 (38.4) | 330 (42.0) | 261 (48.6) | 250 (60.0) | 416 (64.6) |  |

Abbreviations:BMI, body mass index.

**Supplemental Table3|**Predictive ability of several models in external validation.

| Model | AUC | Accuracy | Sensitivity | Specificity | Precision | F1 |
| --- | --- | --- | --- | --- | --- | --- |
| LR | 0.78 | 0.718 | 0.671 | 0.752 | 0.667 | 0.669 |
| SVM | 0.772 | 0.715 | 0.672 | 0.746 | 0.662 | 0.667 |
| GBM | 0.771 | 0.69 | 0.792 | 0.615 | 0.604 | 0.685 |
| NB | 0.716 | 0.667 | 0.633 | 0.692 | 0.603 | 0.618 |
| ANN | 0.772 | 0.704 | 0.718 | 0.693 | 0.634 | 0.674 |
| RF | 0.747 | 0.688 | 0.648 | 0.717 | 0.629 | 0.638 |
